# Simultaneous primary thyroid MALT lymphoma and papillary thyroid cancer

**DOI:** 10.3389/fendo.2024.1372661

**Published:** 2024-05-31

**Authors:** Jianyong Zhang, Jing Wu, Liangquan Sun, Yuan Chen, Zhaoyan Yu

**Affiliations:** ^1^ Department of Vascular and Thyroid Surgery, Guizhou Provincial People’s Hospital, Guiyang, Guizhou, China; ^2^ Guizhou Medical University, Guiyang, Guizhou, China; ^3^ Department of Neurology, The Second People ‘s Hospital of Guiyang, Guiyang, Guizhou, China; ^4^ Department of Breast and Thyroid Surgery, Guiyang Maternal and Child Health Care Hospital, Guiyang, Guizhou, China

**Keywords:** thyroid MALT lymphoma, papillary thyroid cancer (PTC), collision tumor, case report, literature review

## Abstract

The mucosa-associated lymphoid tissue (MALT) lymphoma subtype, specifically extranodal marginal zone B-cell lymphoma, is a rare variant. Within this subtype, primary thyroid MALT lymphoma is an uncommon occurrence. The literature provides limited documentation on thyroid MALT lymphomas, as their prevalence is comparatively lower than in other organ sites. The coexistence of papillary thyroid carcinoma (PTC) and thyroid MALT lymphomas is exceedingly rare. It presents a rare case of primary thyroid MALT lymphoma accompanied by PTC, thyroid lymphoma not being considered before surgery. A 64-year-old female patient, who had been experiencing symptoms related to a substantial thyroid tumor for a duration of three years, she refused to do a needle biopsy before surgery and expressed a preference for surgical resection. Consequently, the patient underwent a total thyroidectomy along with lymphadenectomy of the central compartment. A histological examination subsequently confirmed the presence of papillary thyroid carcinoma (PTC) and mucosa-associated lymphoid tissue (MALT) lymphoma. Due to the favorable response of the MALT lymphoma to local treatment and the absence of metastasis in other organs, no further treatment was administered for the MALT lymphoma following the surgery. Currently, the patient exhibits no signs of tumor recurrence based on ultrasound and laboratory evaluations. We also provide an overview of the clinical findings on PTC and MALT lymphoma patients already reported and discuss the possible treatment strategy.

## Introduction

The global ranking of thyroid cancer incidence places it at fifth position, with a notable increase observed in numerous countries since the 1980s (1). This rise can be attributed to advancements in detection and diagnosis techniques, resulting in a significant upsurge in the detection rates of papillary thyroid carcinoma (PTC) (1). PTC is the predominant type of thyroid cancer, affecting approximately 80% of patients (2). Conversely, extranodal marginal zone B-cell lymphoma of the mucosa-associated lymphoid tissue (MALT lymphoma) subtype is a rare variant primarily found in gastrointestinal tract lymphomas (3). Additionally, the salivary gland, thyroid gland, eye, skin, and thymus gland are frequently identified as the predominant locations (3, 4). Primary thyroid lymphoma constitutes a mere 0.6% to 5% of thyroid tumors (3, 4). The literature scarcely documents thyroid MALT lymphomas due to their comparably lower prevalence in comparison to other organ sites (4). Resulting in a dearth of consensus regarding the diagnosis and treatment of affected individuals, primarily due to its exceptionally low occurrence. Moreover, the coexistence of papillary thyroid carcinoma and thyroid MALT lymphomas is exceedingly rare. A case of concurrent primary thyroid MALT lymphoma and PTC is presented here.

## Case description

**Figure 1:**
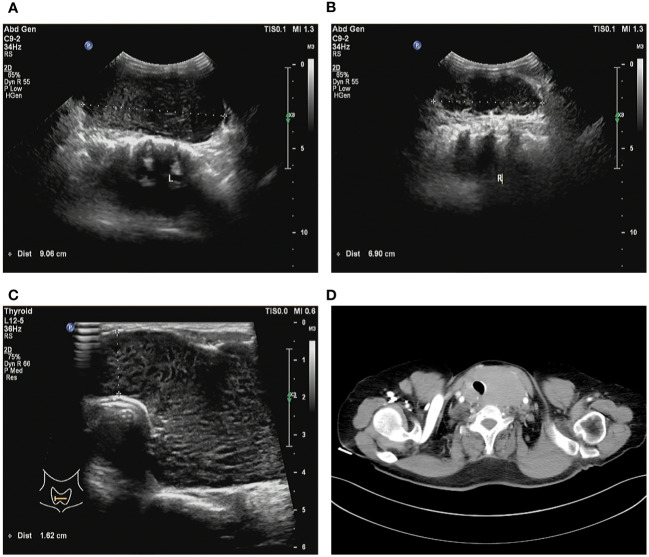
Preoperative imaging images. **(A)** Ultrasound examination detected the left thyroid gland measuring approximately 9 cm. **(B)** Ultrasound examination detected the right thyroid gland measuring over 6 cm. **(C)** The isthmus of the thyroid displayed a thickness of 1.6 cm. **(D)** Computed tomography imaging demonstrates significant enlargement of the thyroid gland, resulting in evident compression and displacement of the trachea.

**Figure 2:**
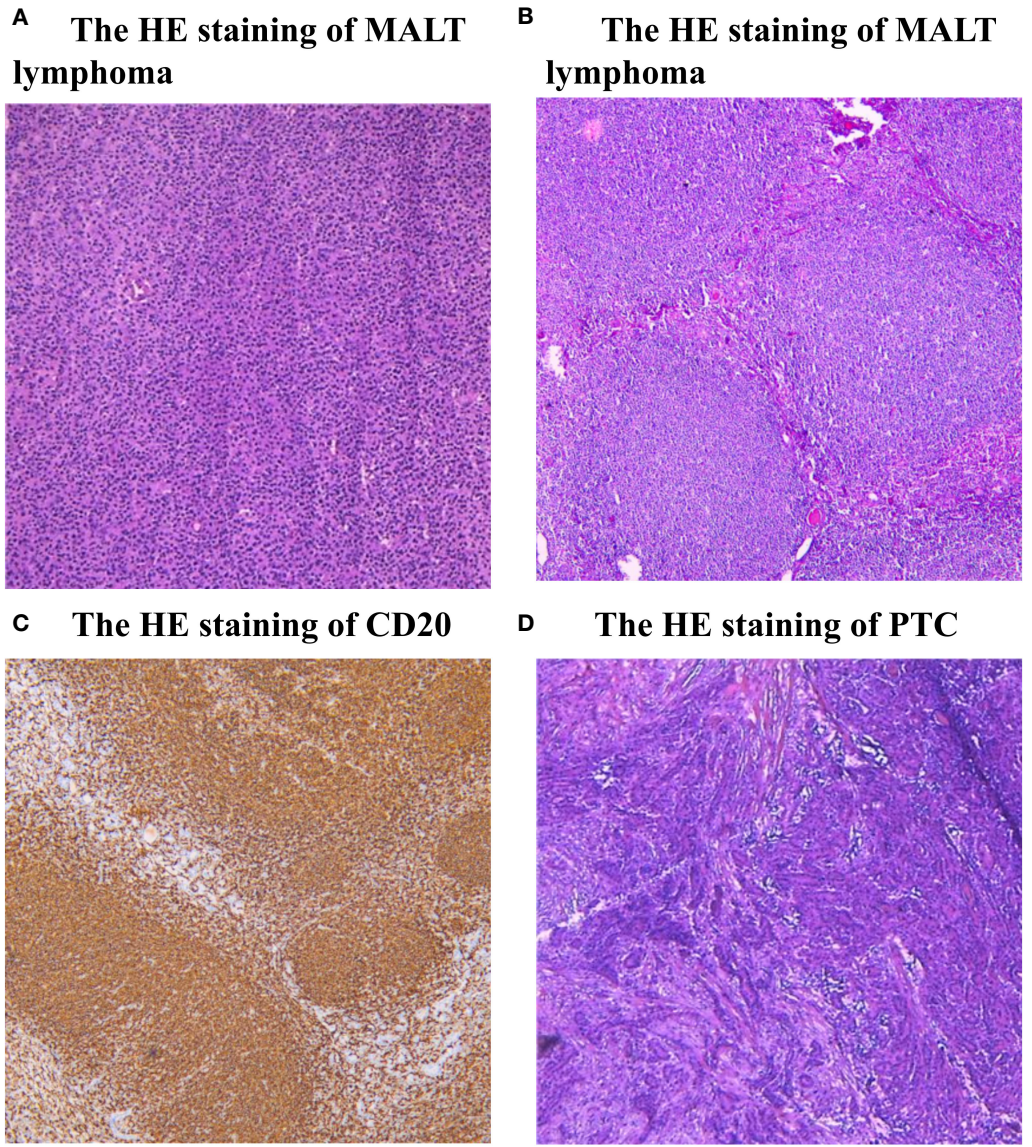
**(A, B)** Histopathological examination (HE) staining of MALT lymphoma. The presence of lymphoproliferative lesions on thyroid glands, atrophy of thyroid follicles, active hyperplasia of a small number of follicular epitheliums. **(C)** Immunohistochemistry (IHC) analysis demonstrated positive expression of CD20. **(D)** Histopathological examination (HE) staining of PTC, there is a typical cancer nest change in the thyroid gland.

A 64-year-old female patient sought evaluation at the vascular thyroid surgery clinic for the purpose of assessing a substantial thyroid tumor that had appeared three years ago. The thyroid tumor was detected, with the left thyroid gland measuring approximately 9 cm and the right thyroid gland measuring over 6 cm. Furthermore, the isthmus of the thyroid displayed a thickness of 1.6 cm (**Figures 1A–C**). The patient had a medical history of Hashimoto thyroiditis and follicular thyroid adenoma and had previously undergone surgical removal of a left thyroid follicular adenoma 16 years ago. Before this surgery, the patient exhibited normal thyroid function, as indicated by normal levels of FT3, FT4, and TSH. The levels of thyroid peroxidase antibodies and thyroglobulin antibodies were found to be elevated (Anti-thyroid peroxidase antibody titers exceeding 600 IU/ml and anti-thyroglobulin antibody titers exceeding 4000 IU/ml), whereas thyroglobulin (0.51 ng/ml) levels were decreased. Based on the results of a blood test, the white blood cell count was 2.57*10^9^/L and the lymphocyte absolute number was 0.39 *10^9^/L. Consequently, the patient exhibited diffuse enlargement of the thyroid gland, indicating a possible association with Hashimoto thyroiditis prior to surgery. It is worth noting that the patient had no history of smoking or habitual alcohol consumption. Laryngoscopy did not reveal any signs of vocal cord paralysis, while ultrasound examination detected diffuse enlargement of the thyroid gland along with a right thyroid nodule, which consider a thyroid cancer. Furthermore, the computed tomography (CT) scan demonstrated significant enlargement of the thyroid gland, resulting in evident compression and displacement of the trachea (**Figure 1D**). As a result of enduring symptoms, the patient expressed a preference for surgical resection. Consequently, the patient underwent an open surgical procedure to completely remove the thyroid gland, with the objective of establishing a conclusive diagnosis and administering suitable treatment. No residual tumor was intraoperatively recognized. Subsequent intraoperative histopathological examination confirmed the presence of lymphoproliferative lesions on both thyroid glands, atrophy of thyroid follicles, active hyperplasia of a small number of follicular epitheliums (**Figures 2A, B**), and the presence of a 0.5 cm papillary thyroid carcinoma on the right thyroid gland based on the pathological findings (**Figure 2D**). We then dissected the central lymph nodes according to Chinese guidelines and our center’s norms, and the pathological results showed no cancer metastases. The postoperative examination of paraffin sections confirmed a notable diffuse proliferation of lymphoid tissue, the formation of lymphoid follicles, and a significant decrease in thyroid follicles on both sides of the thyroid gland. Immunohistochemical analysis revealed a predominant proliferation of B lymphocytes, a reduced follicular dendritic cell network within germinal centers of certain lymphoid follicles, expanded marginal areas, an increased presence of plasma-like differentiated cells, and positive B-cell gene rearrangement. These findings collectively led to the diagnosis of MALT lymphoma. Immunohistochemistry (IHC) analysis demonstrated positive expression of CD20 (**Figure 2C**), CD79, Pax-5, CD19, MUM-1, BCL-2, BCL-6, CD10, CD38 (in plasmoid differentiated cells), CD138 (in plasmoid differentiated cells), CD21 (in follicular dendritic cell network), CD23 (in follicular dendritic cell network), CD30, CD3 (in T cells), CD5 (in T cells), CKpan (in thyroid epithelial cells), and TTF-1 (in thyroid epithelial cells) within the tumor cells of this lesion. The Ki-67 labeling index was determined to be 20%. Additionally, the Immunoglobulin H (IgH) gene exhibited positive expression, while Immunoglobulin kappa (IGK) and Immunoglobulin lambda (IGL) showed negative expression. Additionally, there was no evidence of diffuse large B-cell lymphoma (DLBCL) since the B-cell size was either normal or slightly enlarged (**Figures 2A, B**).

## System reviews the diagnostic assessment, the therapeutic intervention, follow-up, and outcomes of thyroid MALT lymphoma

MALT lymphoma is an indolent and low-grade type of primary thyroid lymphomas (PTL), there is a common clinical symptom of painless enlarging neck swelling with compressive characteristics that can occur progressively or suddenly (5). There is still some debate about the reliability of fine needle aspiration cytology (FNAC) in the detection of PTL, even though it is the gold standard for detecting differentiated thyroid carcinoma (6). While fine needle aspiration and cytology (FNAC) evaluation of specimens is essential for PTL diagnosis, results may be confused with Hashimoto’s thyroiditis (5, 7). Additionally, it is also misdiagnosed as painless subacute thyroiditis by FNAC (5). The management of PTL has incorporated various treatments, such as surgery, chemotherapy, and radiotherapy, but no clear guidelines seem to exist (5). Previous research suggested that a Stage IE (Involvement of the thyroid alone) thyroid MALT lymphoma can be treated with either radiotherapy or surgery alone, but those with a stage greater than or equal to IE MALT lymphoma should receive multidisciplinary treatment to maximize their chances of survival (3, 8). While another scholar revealed that radiotherapy of the involved site is the favored choice for localized MALT lymphoma (9). There are also studies showing that thyroidectomy combined with radiation is also effective (3, 9). If differentiated thyroid cancer is present, surgical removal of the whole thyroid may be a better choice. For outcome, previous data revealed the overall survival rate for MALT lymphoma of the thyroid is 90–96%, and the event-free survival rate is 92% after a 5-year follow-up (10).

## Discussion

Specifically, a collision tumor is a neoplastic lesion composed of cells from different cell populations that form distinct borders (11). For this reason, the present case can be categorized as a collision tumor. It is extremely rare for PTC and MALT lymphoma to coexist. Vicky Cheng et al. reported the first case of a collision tumor between PTC and MALT lymphoma in 2012 (12). MALT lymphoma, a specific type of indolent B-cell lymphoma, exhibits the ability to generate extranodal manifestations. Recent studies have shown that MALT lymphoma constitutes approximately 8% of all B-cell lymphomas, with a notable occurrence rate of 35–50% in the stomach (3). Furthermore, it is frequently observed in the lungs, thyroid, parotid, and eye adnexa. Notably, chronic antigenic stimulation is closely associated with MALT lymphoma, as evidenced by the presence of Helicobacter (H.) pylori infections in up to 80% of patients with gastric MALT lymphoma (3, 13). Thyroid lymphoma constitutes a small proportion, ranging from 0.6% to 5% of all thyroid cancers, whereas MALT lymphoma accounts for approximately 10% of thyroid lymphomas (3, 4). Furthermore, autoimmune disorders have been recognized as potential catalysts or enhancers of mucosa-associated lymphoid tissue (MALT) lymphoma (13). For instance, thyroid lymphoma is frequently linked to Hashimoto’s thyroiditis (HT). The fact that HT is associated with both PTC and MALT lymphoma suggests that patients with HT should be closely monitored for both types of lymphoma (14). Notably, the prognosis for MALT lymphomas is generally more favorable, with 90–96% of patients surviving five years after diagnosis (10). However, the diagnosis and treatment of thyroid lymphoma remain subjects of controversy. Local treatment such as total thyroidectomy or radiation therapy alone is usually effective in treating pure MALT lymphoma (15). Additionally, both radiotherapy and chemotherapy are utilized in the management of recurrence (3). Derringer et al. found no significant association between the type of treatment (solely surgery, surgery with radiotherapy, surgery with chemotherapy, surgery with multimodal therapy) and survival outcomes (16). Therefore, when addressing the combination of PTC and MALT lymphoma, the primary consideration lies in surgical or chemoradiotherapy treatment (10). MALT lymphoma is generally accepted as operable when it is in the early stages, such as what was done in this case. Further large-scale follow-up data are necessary to obtain the best treatment options for PTC and MALT lymphoma.

## Conclusions

Surgical intervention emerges as the preferred approach for managing PTC, while local interventions like total thyroidectomy or radiation therapy alone typically yield satisfactory outcomes in the treatment of pure MALT lymphoma. However, when confronted with the coexistence of PTC and MALT lymphoma, the primary focus should be on surgical treatment, with the potential inclusion of alternative modalities contingent upon the patient’s specific clinical presentation.

## Data availability statement

The original contributions presented in the study are included in the article/supplementary material. Further inquiries can be directed to the corresponding authors.

## Ethics statement

The studies involving humans were approved by Guizhou Provincial People’s Hospital. The studies were conducted in accordance with the local legislation and institutional requirements. The participants provided their written informed consent to participate in this study. Written informed consent was obtained from the individual(s) for the publication of any potentially identifiable images or data included in this article.

## Author contributions

JZ: Data curation, Funding acquisition, Investigation, Writing – original draft, Writing – review & editing. JW: Validation, Writing – original draft, Writing – review & editing. LS: Validation, Writing – review & editing. YC: Formal analysis, Writing – review & editing. ZY: Resources, Validation, Writing – review & editing.
